# Genome-wide expression QTL mapping reveals the highly dynamic regulatory landscape of a major wheat pathogen

**DOI:** 10.1186/s12915-023-01763-3

**Published:** 2023-11-20

**Authors:** Leen Nanchira Abraham, Daniel Croll

**Affiliations:** 1 Laboratory of Evolutionary Genetics, Institute of Biology, University of Neuchâtel, 2000 Neuchâtel, Switzerland; 2 Present address: Institute of Plant Sciences, University of Cologne, Cologne, Germany

**Keywords:** *Zymoseptoria tritici*, Expression quantitative trait mapping, GWAS, Population genetics, Plant pathogens

## Abstract

**Background:**

In agricultural ecosystems, outbreaks of diseases are frequent and pose a significant threat to food security. A successful pathogen undergoes a complex and well-timed sequence of regulatory changes to avoid detection by the host immune system; hence, well-tuned gene regulation is essential for survival. However, the extent to which the regulatory polymorphisms in a pathogen population provide an adaptive advantage is poorly understood.

**Results:**

We used *Zymoseptoria tritici*, one of the most important pathogens of wheat, to generate a genome-wide map of regulatory polymorphism governing gene expression. We investigated genome-wide transcription levels of 146 strains grown under nutrient starvation and performed expression quantitative trait loci (eQTL) mapping. We identified *cis*-eQTLs for 65.3% of all genes and the majority of all eQTL loci are within 2kb upstream and downstream of the transcription start site (TSS). We also show that polymorphism in different gene elements contributes disproportionally to gene expression variation. Investigating regulatory polymorphism in gene categories, we found an enrichment of regulatory variants for genes predicted to be important for fungal pathogenesis but with comparatively low effect size, suggesting a separate layer of gene regulation involving epigenetics. We also show that previously reported trait-associated SNPs in pathogen populations are frequently *cis*-regulatory variants of neighboring genes with implications for the trait architecture.

**Conclusions:**

Overall, our study provides extensive evidence that single populations segregate large-scale regulatory variation and are likely to fuel rapid adaptation to resistant hosts and environmental change.

**Supplementary Information:**

The online version contains supplementary material available at 10.1186/s12915-023-01763-3.

## Background

The control of gene expression is essential for the development and survival of organisms. The foundation of gene regulation is the interaction of regulatory proteins with specific DNA (regulatory) sequences in the coding or non-coding regions of the genome. Transcription factors (TFs) modulate gene expression by binding to transcription factor binding sites in regulatory regions [1, 2]. For instance, TF binding to the promoter near the transcription start site (TSS) helps to initiate transcription by forming a transcription initiation complex [3]. The regulatory region of a gene was thought to be usually located upstream of the TSS. However, comprehensive studies across eukaryotes showed that both the 5′ and 3′- untranslated regions, introns, and even coding regions can act as regulatory sequences [4]. In addition to regulatory sequences, eukaryotic gene regulation is also governed by chromatin structure and histones [5].

Even though core mechanisms of gene expression are conserved across eukaryotes, there is substantial variation in gene expression within species. Individual genotypes can carry adaptive regulatory mutations adjusting gene expression to different environmental cues. In clinical strains of yeasts, promoter variants can cause the upregulation of biofilm suppressor genes and increases pathogenicity [6]. Different consumption rates of aspartic and glutamic acid during the fermentation of yeast hybrids are mediated by the reduced binding affinity of the transcriptional activator protein (Uga3) underpinned by single-nucleotide polymorphisms (SNPs) in the binding region [7]. A synonymous mutation can increase the fitness of *Pseudomonas fluorescens* by increasing gene expression [8]. Hence, understanding how regulatory variation can promote adaptive evolution is important and requires population-scale approaches. However, most evidence relies on a few model organisms such as yeasts [9–11], *Drosophila* [12, 13], humans [14–16], or *C. elegans* [17] and we lack comprehensive studies of regulatory variation in many ecologically relevant species.

Mapping of regulatory mutations in model organisms generated fundamental insights into the extent of regulatory polymorphism and gene regulatory networks. SNPs, insertions, deletions, copy-number variants, and transposable elements (TE) can act as a genetic variant [9, 18, 19]. Genetic variants associated with mRNA level variation are called expression quantitative trait loci (eQTLs). eQTLs can be located in regulatory sequences, such as promoters, enhancers, and splice sites, or gene elements such as 5′ or 3′ UTR, exons, and introns. Most eQTLs are acting in *cis* on the neighboring genes. But eQTLs can also be in genes typically encoding regulatory proteins that interact with *cis*-regulatory sequences on the same or different chromosome (i.e., *trans-*eQTLs) [11, 20, 21]. Species differ in their organization of regulatory regions. For instance, in yeasts, regulatory variants are enriched upstream of the TSS (i.e., the promoter) and 3′UTR whereas in human and *Drosophila* SNPs located near the TSS and in the 5′UTRs are more likely to be eQTLs [9, 22]. *Cis*-eQTLs tend to be more common and have larger effect sizes on gene expression variation than *trans-*eQTLs. Exceptions include for example the plant pathogenic fungi *Botrytis cinerea* and *Coprinopsis cinerea* [23–25]. Plant pathogens often harbor also polymorphism for gene expression during pathogenesis with likely strong selective advantages [26–29].

Plant pathogens cope with multiple environmental stressors over the life cycle with well-timed sequences of regulatory changes. During plant infection, pathogens tightly control gene expression to circumvent recognition by the host [30–33]. Pathogens secrete effectors (i.e., small-secreted proteins) at the onset of host infection serving to manipulate the host cell biology, repress host immune responses, or shield the pathogen to support growth and colonization [34–37]. Because of their prime role during infection, effector gene expression is highly upregulated upon contact with the host [30]. Carbohydrate-active enzymes (CAZyme) are a second major component of fungal pathogenicity and serve to break down plant cell wall components to aid host colonization. *Zymoseptoria tritici*, a major pathogen of wheat, shows evidence for intra-specific regulatory variation and high genetic diversity from single field populations to continents [38, 39]. Populations show a rapid decay in linkage disequilibrium which helps increase the power of mapping approaches and narrows down associations [40–42]. The insertion of a TE led to the downregulation of a recognized effector gene and enabled the strain to evade recognition by the host [43]. Similarly, differences in the production of melanin were shown to be controlled by epigenetic regulation of nearby TEs underlining the significance of regulatory variants associated with gene expression variation within pathogen populations [44, 45]. TE-associated polymorphisms and effects on gene expression are widespread in the genome and underpin phenotypic trait variation even within single field populations [46, 47]

In this study, we map regulatory variation at the genome-wide scale in the fungal pathogen of wheat *Z. tritici* under controlled culture conditions. We associate variation in gene transcription levels with genetic variation from strains collected from a highly diverse field population. We assess *cis* and *trans-*regulatory variation segregating for different gene functions and genomic locations. Furthermore, we test whether different functional gene categories important for pathogenesis show differences in the level of segregating regulatory variation. Finally, we analyze contributions of regulatory variations on phenotypic trait variation within the species.

## Results

### Polymorphism analyses of the mapping population

**Fig. 1:**
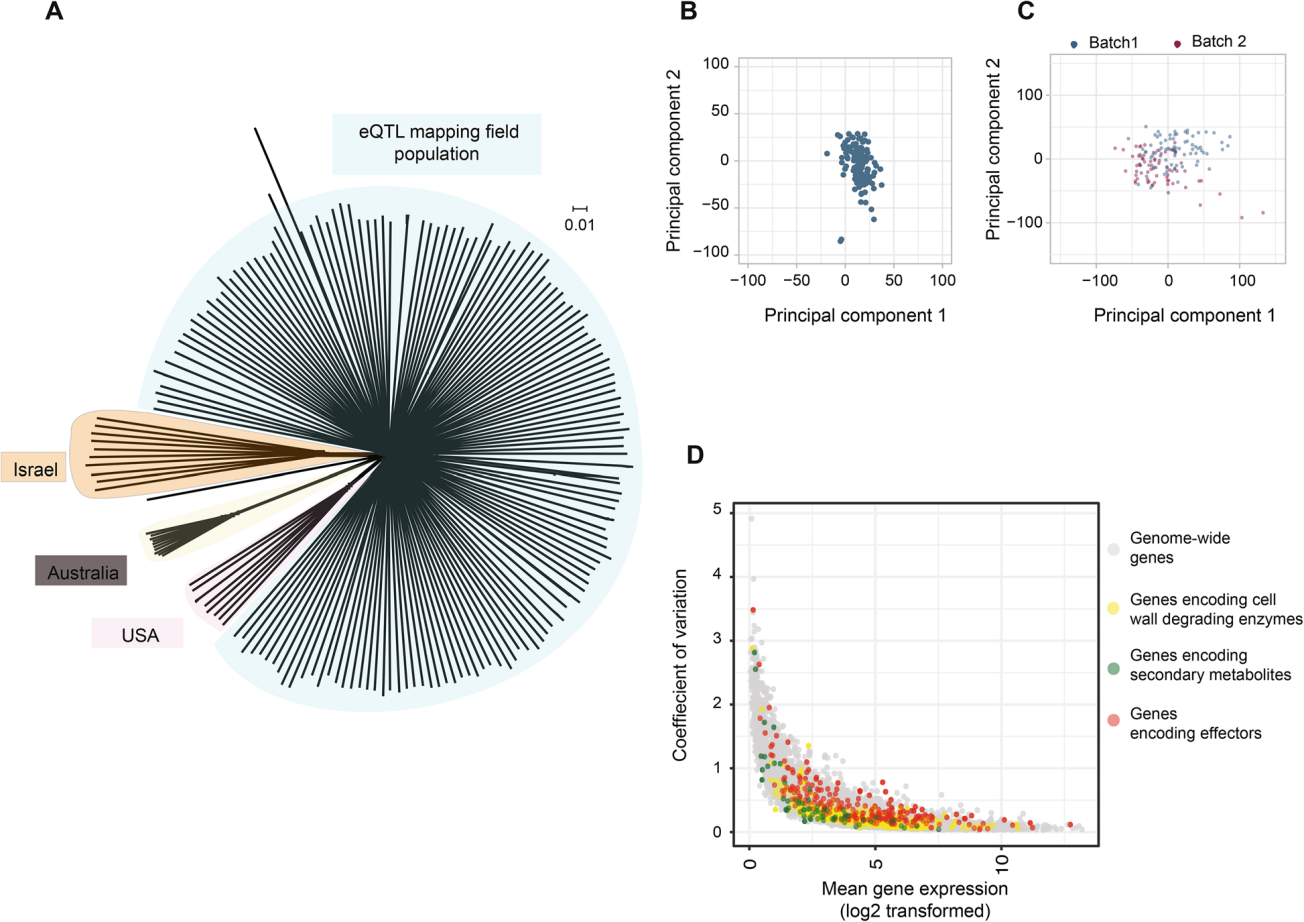
Genetic diversity of the mapping population. **A** Unrooted phylogenetic network of the mapping population and representative isolates from a global collection of *Z. tritici* populations (*n* = 146). **B** Principal component analysis of genetic variation within the mapping population. **C** Principal component analysis of gene expression in the mapping population. Colors differentiate culture batches. **D** Coefficient of gene expression variation against the RPKM normalized values of gene expression (log2-transformed). Colors indicate different gene categories relevant for pathogenesis

We analyzed 146 *Z. tritici* isolates from a field population in Switzerland using whole-genome and transcriptome sequencing [38, 46]. Variant calling on whole-genome sequences of the mapping population identified 543,046 SNPs and 39,356 indels after quality filtering. The isolates were highly diverse compared to the worldwide diversity of the pathogen (Fig. 1A). We identified a strong skew of minor allele frequencies towards rare variants consistent with a large, recombining population (Additional file 1: Fig. S1). A principal component analysis of genetic variants showed no evidence for population substructure among the analyzed isolates with only 2% of the cumulative variance explained by PC1 (Fig. 1B, Additional file 1: Fig. S2). We quantified genome-wide transcription levels of the 146 *Z. tritici* isolates under nutrient starvation conditions (Vogel’s Medium N (Minimal) [48] modified as ammonium nitrate replaced with potassium nitrate and ammonium phosphate [49] without sucrose and agarose). The starvation condition stimulates mycelial growth and induces transcriptomic changes resembling early plant infection stages [43]. A PCA of gene expression showed minor clustering according to culturing and RNA collection batches and cumulative variance explained by PC1 was around 10% (Fig. 1C, Additional file 1: Fig. S3). The population showed high expression variation between different gene categories important for pathogenesis (Fig. 1D). To account for heterogeneity in the mapping population, we included the first PC from the genetic substructure analysis, as well as PCs 1–10 from the gene expression analyses as covariates in the genome-wide mapping (Additional file 1: Fig. S3). To correct for the batch effect in gene expression, we included the RNA sequencing batch as a random effect in the association mapping analyses (Fig. 1C).

### Genome-wide regulatory variation

**Fig. 2:**
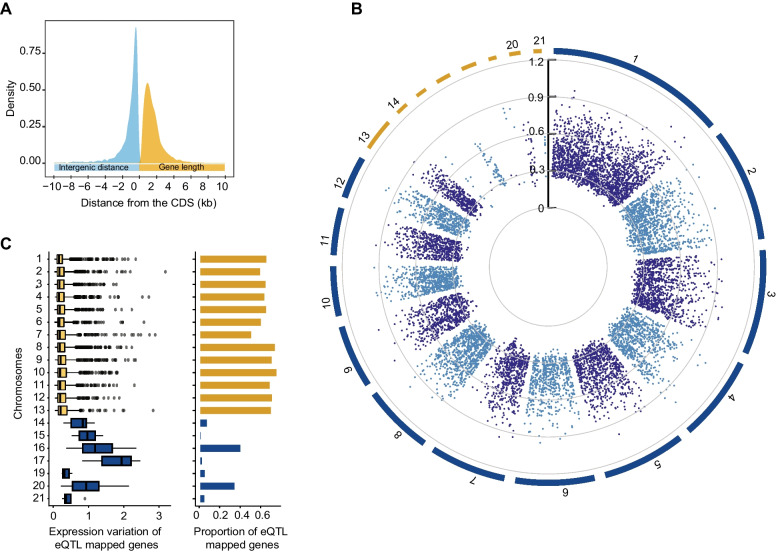
Genome-wide distribution of mapped *cis*-eQTLs. **A** Genome-wide distribution of gene length and intergenic distances. **B** Genome-wide distribution of mapped *cis*-eQTLs (*n* = 11,083). The scale inside indicates the effect size of the eQTLs (points represent individual *cis*-eQTLs and different colors distinguish chromosomes). **C** Distribution of expression variation and proportion of genes with an eQTL in core (blue) and accessory (yellow) chromosomes. Gene expression on accessory chromosomes is normalized for chromosome presence-absence in the population

To identify *cis*-regulatory variation, we associated the effect of polymorphisms around TSS to gene expression variation of the gene. The *cis* window comprises both 5 kb from the TSS upstream and downstream. In the compact genome of *Z. tritici*, this *cis* window covers most of the gene length (mean gene length ~ 1.6kb) and avoids significant overlap with neighboring genes (mean intergenic distance ~ 1.7kb; Fig. 2A). We observed that the majority of the significant *cis*-eQTLs mapped near the 10kb window around TSS even with an increase in cis window to 25kb from the TSS (Additional file 1: Fig. S4). We identified 11,083 *cis*-eQTLs (FDR > 5%) across the genome with 65.3% (*n* = 7396) of the genes showing at least one *cis*-eQTL (Fig. 2B, Additional file 2: Table S1). Among genes with a mapped *cis-*eQTL, 61.2% of the genes show a single *cis*-eQTL, a further 29.3, 7.7, and 1.3% show two, three, and four *cis-*eQTL, respectively (Additional file 1: Fig. S5). The proportion of genes with at least one mapped eQTL was substantially higher on core chromosomes compared to accessory chromosomes (Fig. 2C).

### Contribution of gene features to regulatory variation

**Fig. 3:**
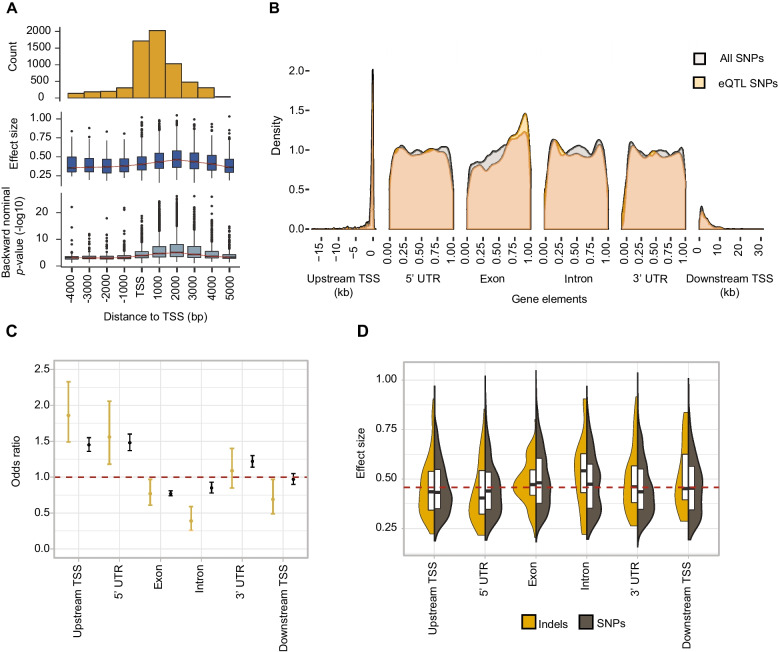
Localization of *cis*-eQTLs across gene elements. **A** Density of mapped eQTLs in *cis* windows. **B** Distribution of *cis*-eQTLs relative to different gene elements. 5′, UTR, exon and intron positions are shown scaled to 1. Upstream/downstream TSS positions are shown in kb. **C** Enrichment of *cis*-eQTLs in gene elements (odds ratio of observed over expected). Color distinguishes polymorphism types at the eQTL (yellow: indel; brown: SNP). **D** The effect size of high-impact eQTLs across gene elements

We analyzed whether *cis-*eQTL were enriched in specific gene elements. We observed varying densities of *cis*-eQTLs in the coding sequence, 3′, 5′UTR, intron, upstream of TSS, and downstream of TES respectively (Fig. 3A, B) with a higher density of *cis*-eQTLs in the coding sequence and an increase in density towards the 3′ end. To account for variation in SNP densities across the gene element, we assessed the enrichment of SNPs being mapped as an eQTL in different gene elements. We found an enrichment of *cis*-eQTL upstream of the TSS, 5′ UTR, and 3′UTR, regardless of the variant category (Fig. 3C). In contrast to the enrichment pattern, the effect size is higher for *cis*-eQTLs mapped in exons and introns. We found no significant difference in effect size for synonymous and non-synonymous mutation in exonic *cis*-eQTLs (Additional file 1: Fig. S6). *Cis*-eQTLs mapped to indels within introns showed a higher effect size than other *cis*-eQTLs (Fig. 3D).

**Fig. 4:**
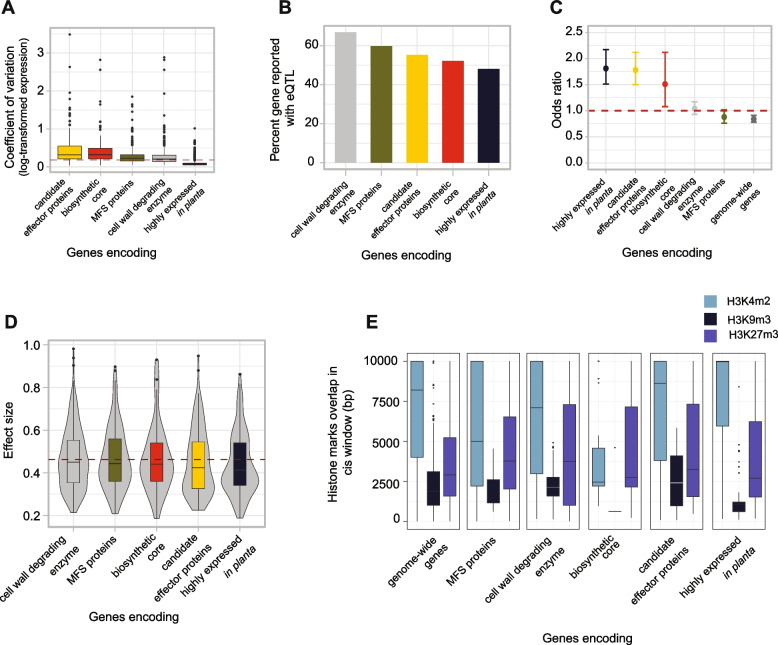
The proportion of *cis*-eQTLs identified across gene categories. **A** Expression variation of gene categories across gene categories. **B** Percentage of genes reported with at least one eQTL across gene categories. **C** Enrichment of cis-eQTLs across gene categories. **D** Effect size of high-impact eQTLs across gene categories. The red line indicates the cis-eQTL effect size of genome-wide genes. **E** Overlap of three different histone methylation marks in cis windows of different gene categories

Furthermore, we assessed SNPs mapped as *cis*-eQTLs between different gene categories encoding essential protein functions for plant pathogens to successfully infect and exploit host tissue. We selected gene categories encoding cell wall-degrading enzymes (CAZymes), genes encoding predicted effectors (predicted EffectorP v 2.0 [50]), secondary metabolite gene clusters [51], major facilitator superfamily (MFS) genes, and genes highly upregulated during plant infection [31]. We analyzed the coefficient of gene expression variation (CV) across these gene categories and found that genes encoding effectors have higher CV than other gene categories whereas genes highly expressed in planta showed the least expression variation (Fig. 4A). The proportion of genes with at least one mapped eQTL is lower for genes with high expression in *planta* (48%) followed by core biosynthetic genes of secondary metabolite gene clusters (54%), genes encoding candidate effectors (55%), genes encoding MFS transporters (62%) and genes encoding CAZymes (67%) (Fig. 4B). Highly expressed genes in planta infection are more likely to have at least one *cis*-eQTL (95% confidence interval; Fig. 4C) but with comparatively lower effect size (Fig. 4D). We hypothesize that epigenetic regulation might be more prevalent than regulatory polymorphism in genes encoding effectors and highly expressed genes during plant infection. To test this, we investigated the coverage of different histone marks in the *cis* window of these gene categories in the reference genome. We observed a higher coverage of euchromatin histone marks H3K4m2 in the window of genes encoding effectors and genes reported with high expression i*n planta* compared to other gene categories (Fig. 4E). H3K4m2 histone marks tend to be involved in the regulation of gene expression, which supports the hypothesis that gene categories mapped with low effect size regulatory *cis*-eQTLs are more likely regulated by epigenetics rather than *cis*-eQTL variants.

### Networks of genes regulated by trans-eQTLs

**Fig. 5:**
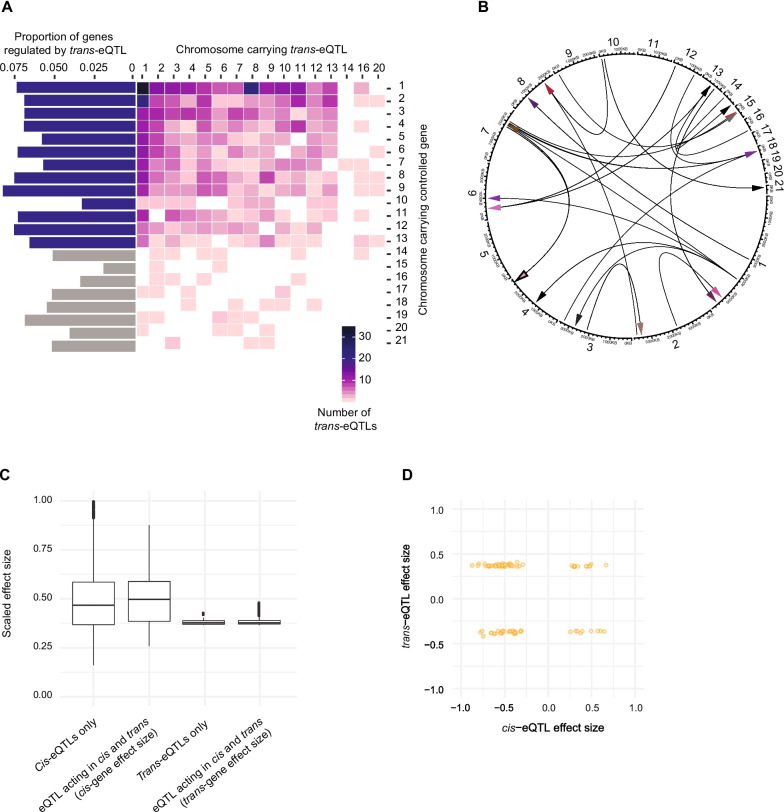
Genome-wide distribution of mapped trans-eQTLs. **A** Distribution of mapped *trans*-eQTLs on core and accessory chromosomes (retained following approximate pass). Barplots on the right indicate the proportion of genes categorized by chromosomes regulated by *trans*-eQTLs. **B** Distribution of *trans*-eQTLs on core and accessory chromosomes. **C** The effect size of overlapping pairs of *cis* and *trans*-eQTLs. **D** Magnitude and direction of gene expression changes for overlapping pairs of *cis* and *trans*-eQTLs. Effect sizes > 0 represent a positive association of the alternative allele with expression and effect sizes < 0 represent a decrease in gene expression with the alternative allele at the eQTL

We performed *trans-*eQTL mapping to identify regulatory variants associated with the expression of distal genes using expression variation among 146 individuals. The full permutation pass with stringent criteria identified 20 genes regulated by *trans-*eQTL with high confidence whereas the approximation pass with less multiple testing burden on *trans-*eQTLs reported 843 genes with at least one *trans-*eQTL (Additional file 2: Table S2, S3). The *trans-*eQTLs from the approximate pass were used to reconstruct genome-wide pattern of *trans-*eQTL occurrence. We found that polymorphisms on core chromosomes are more likely to influence genes on core chromosomes than accessory chromosomes, and *trans*-eQTL identified on accessory chromosomes are more likely to influence genes on core chromosome (Fig. 5A). In contrast to the findings for *cis*-eQTL occurrences, the proportion of genes mapped with at least one *trans-*eQTL does not differ between core and accessory chromosomes (Fig. 5A). We also observed multiple *trans*-eQTLs from different chromosomes regulating a single gene implying a complex network of gene regulation spanning different chromosomes (Additional file 2: Table S3). An interesting locus reported as a *trans*-eQTL is a polymorphism in an exon of the gene 10_00067 located on chromosome 10 associated to the expression of the gene 21_00018 on chromosome 21 (Fig. 5B). Both genes are predicted to be encoding alpha-tubulin suggesting coregulation of alpha-tubulin genes in the genome. A small proportion of *trans-*eQTLs (83 of 843) were found to be overlapping with *cis*-eQTLs. The effect size of eQTLs mapped with *cis* and *trans* effects is higher than the effect size of eQTLs having only a *cis* or *trans* effect (Fig. 5C). These overlapping pairs are possibly explained by functional links between the *cis* and *trans* linked genes. Overlapping *cis* and *trans*-eQTLs can act in opposite direction compensating their effects and stabilize gene expression levels [52]. Among overlapping *cis/trans*-eQTLs, 46 are regulating the expression of *cis* and *trans* genes in the same direction, and 42 in opposite direction (Fig. 5D).

### Trait-associated SNPs are overrepresented in cis-regulatory variants

**Fig. 6:**
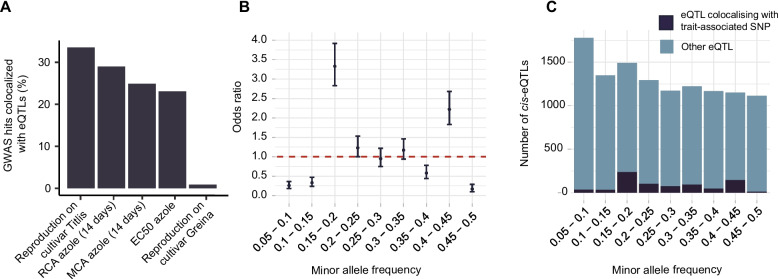
Colocalization of eQTLs with GWAS-associated SNPs. **A** Percentage of phenotypic trait-associated SNPs colocalized with eQTLs. MCA: mean of colony area; RCA: ratio of colony area; EC50: Half-inhibitory concentration. **B** Enrichment of phenotypic trait-associated *cis*-eQTLs across the minor allele frequency spectrum. **C** Number of overlapping phenotypic trait-associated SNPs and *cis*-eQTLs in different minor allele frequencies

**Fig. 7:**
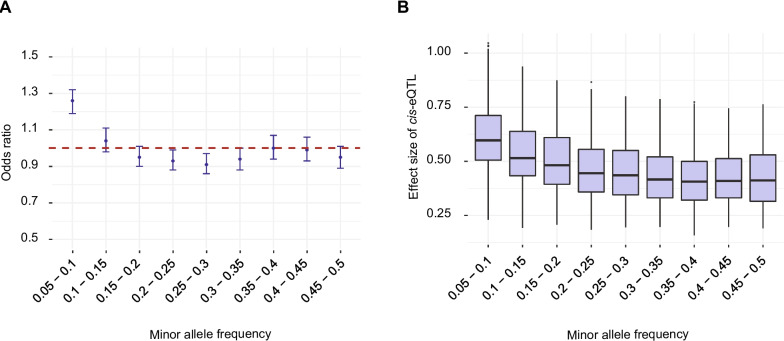
Allele frequency spectrum of *cis*-eQTL variants in the mapping population (*n* = 146). **A** Enrichment of *cis*-eQTLs across minor allele frequency categories. **B** Effect size of *cis*-eQTLs across minor allele frequency categories

How associated polymorphisms influence phenotypic trait variation remains often unknown. We performed analyses linking known variants associated with virulence and fungicide resistance of the pathogen to mapped *cis*-eQTLs [53–55]. Trait-associated SNPs were enriched in *cis*-regulatory variants compared to the genomic background with an odds ratio of 20.82 (95% confidence interval 19.16–22.6). Among all analyzed traits, SNPs associated with azole fungicide resistance and reproduction on a wheat cultivar showed the highest degrees of colocalization with mapped *cis*-eQTLs (Fig. 6A, Additional file 1: Fig. S7). Furthermore, moderately frequent regulatory variants (minor allele frequency 0.15–0.2 and 0.4–0.45) showed the highest overrepresentation of colocalized trait-associated SNPs (Fig. 6B,C). To estimate selection pressure acting on regulatory variants, we assessed potential enrichment of mapped eQTLs for rare or common variants globally. C*is*-eQTLs with low frequency variants were most overrepresented (Fig. 7A). We also observed an inverse relationship between the effect size of the *cis*-eQTL and the minor allele frequency of the top associated SNP (Fig. 7B). Hence, low frequency variants show the strongest effects on gene expression overall. A skew towards low frequency of high-impact regulatory variants is consistent with purifying selection acting against regulatory variation in the population.

### Regulatory architectures of virulence and fungicide sensitivity-associated genes

The eQTL mapping revealed a complex regulatory architecture of previously characterized genes important for the pathogenesis and fungicide resistance (Table 1, Additional file 2: Table S4). The expression of small-secreted proteins is coordinated by transcriptional factors and epigenetic regulation. ZtWor1 is an important transcriptional regulator for the expression of small-secreted proteins [56]. We found a *trans*-eQTL in a gene encoding the protein arginine methyltransferase 5 (PRMT5) associated with the expression of the transcriptional regulator ZtWor1 [57]. Coordinated expression of methyltransferases and the transcriptional regulator of small-secreted proteins may underpin the epigenetic regulation of genes encoding effectors.

**Table 1:** Identification of genes nearby *trans*-eQTL loci as well as the gene controlled by the *trans*-eQTL with putative functions in virulence or fungicide sensitivity

Gene nearby* trans*- eQTL	Functional annotation of gene nearby *trans*- eQTL	Gene controlled by *trans*-eQTL
9_00268	Gene encoding the protein arginine methyltransferase 5 (PRMT5):	*ZtWor1* (8_00107)
7_00175	Gene encoding a fungal trichothecene efflux pump	Gene encoding the necrosis-inducing protein NPP1 (13_00229)
12_00203	Gene encoding the ABC transporter MgATR2	Gene encoding an MFS (1_01912)

Secondary metabolites also play a pivotal role in fungal pathogenesis [58]. A polyketide synthase cluster on chromosome 13 includes a gene encoding the necrosis-inducing protein NPP1 important for virulence in many fungal pathogens even though functional characterization in *Z. tritici* revealed no virulence association [59]. We identified a polymorphism in a gene encoding a fungal trichothecene efflux pump (7_00175) associated with the expression of the gene encoding the necrosis-inducing protein (NPP1; 13_00229). The association supports the idea that the trichothecene efflux pump acts as a transporter in concert with the PKS gene cluster. Azole fungicide sensitivity in *Z. tritici* can be mediated by multiple mechanisms including the efflux of fungicides by specific transporter such as ATP-binding cassette (ABC) transporters [60]. We identified an association of polymorphisms in the gene encoding the ABC transporter MgATR2 within a gene encoding an MFS, which is a superfamily of membrane transporter proteins. These links underline that ABC transporter efflux functions may contribute to the variation in azole fungicide sensitivity in populations.

## Discussion

We generated the first genome-wide map of regulatory variants underpinning variation in gene expression in a fungal plant pathogen. The majority of all genes in the genome segregated at least one *cis*-eQTL in the mapping population derived from a single field site. Variants upstream of the TSS and untranslated regions were more likely associated with expression variation but with lower effect size. Pathogenicity-related genes were less likely associated to eQTLs consistent with epigenetic regulation playing a prominent role. Phenotypic trait variation is likely governed substantially by gene expression variation.

We successfully identified at least one *cis*-eQTL for two-thirds of all genes indicating how broadly regulatory variation segregates within populations. Genes lacking associated regulatory variants possibly reflect statistical limitations in detecting small effect sizes or the relevant variants were fixed in the analyzed population. Moreover, we assessed transcriptome variation from fungi growing as mycelium in conditions inducing nutrient starvation stress. Under these conditions, *Z. tritici* modulates the transcriptome to resemble early plant infection stages [43]. Hence, genes repressed in any genetic background under the tested condition are not accessible to eQTL mapping in our analyses. Assessing the transcriptome under controlled infections is feasible; however, variation in infection severity among genotypes may confound eQTL mapping because of the non-controlled environmental effects during infection. *Cis*-eQTLs reported from other intra-species mapping studies varied from 20% of genes in 85 diverse yeast strains [20], 28% of genes in the *D. melanogaster* genetic reference panel (DGRP) of 39 inbred lines [12], 13% of genes in *A. thaliana* populations [61], 6.7% of genes in humans with 270 individuals from multiple populations, and 31% of the genes in 801 European and 1032 African Americans [62–64]. Mapping studies differed by panel size and genetic diversity of the mapping population. The comparatively smaller population size used for mapping might explain the lower proportion of *cis*-eQTLs in the populations of *A. thaliana* and DGRP lines of *D. melanogaster* (a representative sample of naturally segregating genetic variation). The small number of eQTLs mapped in the comparatively large mapping population of *S. cerevisiae* may stem from slow linkage disequilibrium decay. Population genomics studies in *Z. tritici* reported that ~ 90% of the global genetic variation is found within single populations and with few indications of bottlenecks [38, 39, 65–67]. Hence, *Z. tritici* populations are highly amenable to the efficient mapping of *cis*-eQTLs.

A substantial minority of all genes were mapped with multiple eQTLs consistent with the high-resolution of the panel and complexity of *cis*-regulatory variation in the species. The primary regulatory variant for each gene was located close to the TSS and the effect of regulatory variants on gene expression was inversely proportional to the distance of the variant from the TSS. Hence, dominant regulatory variants are likely binding sites and promoters proximal to TSS. Indels in introns, exons, and 3′ UTR had stronger effects on gene expression compared to SNPs. Indels are more likely to disrupt the integrity of splice sites, RNA-binding protein motifs in 3′UTR and are, hence, more likely causal variants for gene expression variation than SNPs [68]. Indel mutations at DNA binding sites can strongly reduce the binding affinity of DNA-binding proteins such as TFs, whereas the degenerate nature of DNA binding sites can balance out more easily the effects of point mutations [69]. Intronic polymorphisms had stronger effects on gene expression variation but were less likely to be identified as *cis*-eQTLs, which is consistent with stronger selective constraints on intronic polymorphisms. In plants such as *A. thaliana*, the larger introns can likely more effectively buffer deleterious effects of indel mutations [70]. Also, polymorphism in introns may result in alternatively spliced mRNA and functionally diverse proteins with possible phenotypic consequences [71]. The generalist plant pathogen *Sclerotinia sclerotiorum* was found to accumulate alternative transcript isoforms upon infection depending on the host identity, which could indicate alternative splicing events are taking place upon infection to generate functionally diverse secreted proteins [72]. Hence, the strength of constraints on regulatory or splice variants in coding sequences has likely consequences for the evolvability of gene regulation within the species.

Gene regulatory polymorphisms were unevenly distributed among functional categories of genes. eQTLs were underrepresented in genes encoding functions important for pathogenesis, i.e., effectors and secondary metabolites. This observation may stem from unequal success in variant calling steps since genes encoding effector proteins tend to localize in the vicinity of TEs [73]. The repetitive nature of TEs may prevent the identification of reliable SNPs, which in turn could lead to the underestimation of *cis*-eQTLs. In contrast to the lower proportion of genes mapped with eQTLs, the likelihood of a variant being called an eQTL is higher for variants close to the genes encoding effectors, secondary metabolites, and genes highly expressed during plant infection. However, effect sizes of these *cis-*eQTLs were comparatively low to eQTLs for other gene categories. This could suggest that gene regulation for pathogenicity-associated genes tends to be mediated by epigenetics rather than *cis*-regulatory elements. Epigenetic gene regulation can form long-range chromatin interactions mediated by chromatin state changes and can regulate the expression of the target gene from hundreds of kilobases away. Hence, silenced TEs could cause co-silencing of effectors independent of detectable eQTLs. These chromatin interactions are physical interactions of DNA mediated by histone methylation marks and do not require nucleotide variants [74]. This observation suggests the involvement of epigenetic regulation as a major player in effector gene regulation consistent with studies showing effector expression being influenced by TEs [43, 46].

Our *trans-*eQTL mapping identified only few genes associated with regulatory variation compared to *cis*-eQTLs. A similar observation was reported in humans (0.3% of the genes associated with *trans-*eQTLs) [75] and recombinant inbred lines of *D. melanogaster* [76], whereas a study of 1012 yeast segregants from a cross between a laboratory and a wine strain reported almost all the expressed genes as having at least one *trans-*eQTL [77]. A lower number of genes mapped with *trans-*eQTLs than *cis*-eQTLs might be due to the lower mapping power resulting from the high multiple testing burden in *trans-*eQTL analyses. Unlike the mapping studies in *S. cerevisiae* [23] and maize [78], we identified no *trans-*regulatory hot spots (i.e., *trans-*eQTL regulating large gene sets). We expected core and accessory chromosomes of *Z. tritici* to show regulatory links; however, we found that core chromosome genes were almost exclusively regulated core chromosome *trans-*eQTLs. This compartmentalization is consistent with distinct organizations of core and accessory chromosomes. Studies on the filamentous fungus *Epichloë festucae* [79] and *D. melanogaster* [80] both underlined that the 3D structure of the genome can mediate transcription and regulate gene expression. Hence, the *Z. tritici* 3D chromosomal conformation may allow for only few core-accessory chromosome links.

The *trans-*eQTLs mapped for virulence and fungicide sensitivity-associated genes suggested the existence of complex regulatory networks. The *trans-*regulatory polymorphism in the gene encoding a methyltransferase (PRMT5) is associated with expression variation of the transcription factor known as a positive regulator of major virulence genes in the pathogen. PRMT5 mediates the methylation of histone arginine and plays an important role in chromatin dynamics [57]. The coordinated regulation of histone methyltransferase and the regulator of virulence genes highlights the importance of the epigenetic regulation layer of effector gene regulation. The genes encoding secondary metabolites tend to colocalize in clusters containing one or more core biosynthetic genes, accessory genes, major regulators, and transporters [81, 82]. In our *trans-*eQTL mapping, we identified a polymorphism in an exon of the gene encoding a trichothecene efflux pump and regulating the expression of the gene encoding the necrosis-inducing protein (NPP1). NPP1 is also a predicted effector suggesting that secondary metabolite gene clusters may be capable of coordinating gene regulation outside of the cluster. Such effects may include the regulation of transporters relevant for the transport of the secondary metabolite.

## Conclusions

Gaining a mechanistic understanding how polymorphism identified from phenotype-genotype association mapping contributes to phenotypic variation is often challenging. Colocalization of eQTLs and trait-associated SNPs enables more focused hypothesis-testing. Our colocalization approach using SNPs associated with virulence and fungicide sensitivity identified that phenotypic trait-associated SNPs were enriched for regulatory polymorphisms with a quarter of the SNPs colocalizing with mapped *cis*-eQTLs. This underlines that regulatory polymorphism is likely playing a major role in phenotypic trait variation. Hence, recent adaptation of the pathogen to hosts and the environment may have been achieved with substantial contributions from regulatory polymorphisms. Ascertaining standing variation for genetic and regulatory variants enables more precise predictions of the evolutionary potential of species.

## Methods

### Library preparation, genome, and transcriptome sequencing

Isolates of *Z. tritici* were collected from an experimental wheat field planted with different cultivars [38, 83] and grown for 10 days in yeast-sucrose broth (YSB) at 18°C. Total genomic DNA was extracted using the QIAGEN DNAeasy Plant Mini Kit and the Illumina library was prepared using a TruSeq Nano DNA Library kit (Illumina, Inc.). Libraries with an insert size of ~ 550 bp were sequenced for a read length of 100 bp in paired-end mode on a HiSeq 4000 at the iGE3 sequencing platform (Geneva, Switzerland). For RNA sequencing, the same isolates were cultured in a Vogel’s Medium N (Minimal) [48] modified as ammonium nitrate replaced with potassium nitrate and ammonium phosphate [49] without sucrose and agarose to induce hyphal growth [84]. Total RNA was isolated from the filtered mycelium after 10–15 days using the NucleoSpin® RNA Plant and Fungi kit. RNA concentration and integrity were checked using a Qubit 2.0 Fluorometer and an Agilent 4200 TapeStation System, respectively. Only high-quality RNA (RIN > 8) was used to prepare TruSeq stranded mRNA libraries with a 150 bp insert size and sequenced on an Illumina HiSeq 4000 in the single-end mode for 100 bp.

### Variant calling and filtering

DNA sequences were checked for quality using FastQC version 0.11.5 [85] and trimmed with trimmomatic version 0.36 [86] to remove adapter sequences and low-quality reads with parameters ILLUMINACLIP: TruSeq3-PE.fa:2:30:10 LEADING:3 TRAILING:3 SLIDING WINDOW:4:15 MINLEN:36. Trimmed sequences were aligned to the *Z. tritici* reference genome of IPO323 [87] and mitochondrial sequence (accession EU090238.1) using Bowtie2 version 2.3.4.3 [88] with the option –very-sensitive-local. Aligned sequences were used for variant calling with the HaplotypeCaller integrated in the Genome Analysis Toolkit (GATK) v. 4.0.11.0. [89]. SNPs and indels were separated using the SelectVariants tool. SNPs and indels were quality filtered with the VariantFiltration tool. We retained SNPs with QUAL > 1000, AN = 20, QD > 5.0, MQ > 20.0, as well as ReadPosRankSum, MQRankSum, and BaseQRankSum between − 2.0 and 2.0. Variants passing the quality filtration were further filtered to remove multiallelic sites using the bcftools (version 1.9) –norm option. After that, the variants were filtered to keep only sites genotyped in at least 90% of the individuals and rare variants (< 5%) were removed using VCFtools [90] and the –max-missing option and bcftools (version 1.9) [91] -q 0.05: minor option.

### RNA-seq analyses

RNA-seq data were checked for quality using FastQC (version 0.11.5) [85] and trimmed with trimmomatic version 0.36 [86] to remove adapter sequences and low-quality reads with parameters: ILLUMINACLIP: TruSeq3-SE.fa:2:30:10 LEADING:3 TRAILING:3 SLIDING WINDOW:4:15 MINLEN:36. Trimmed sequences were aligned to the *Z. tritici* reference genome of IPO323 [87] using HISAT2 [92] (version 2.1.0) with the parameter “–RNA-strandedness reverse”.

### De novo transcriptome assembly and UTR prediction

For de novo transcriptome assembly, we used RNA-seq datasets generated for the reference isolate IPO323 from different plant infection stages (1, 4, 9, 14, and 21-days post infection) and culture media (Czapek-Dox broth, potato dextrose broth) available on NCBI (accession numbers: ERS684130-37, ERS684123-29, ERS683735-40, ERS6837343). We used also Illumina RNA-seq reads generated from minimal media grown following the above-described methods. For in planta RNA-seq data, reads were filtered by aligning to the IPO323 reference genome [87] to remove transcripts from wheat. Trinity (version v2.8.3) [93] was used for de novo transcriptome assembly using the –jaccard_clip parameter to reduce the occurrence of fused transcripts in dense fungal genomes. We performed de novo assembly using different sets of RNA-seq samples including up to a maximum of ~ 5 million reads per dataset. Next, we used GenomeThreader version 1.7.1 [94] to align de novo assembled transcripts to the reference genome requiring a minimum alignment score of 0.98. Along with de novo assembled transcripts, we used Iso-Seq reads generated for IPO323 to improve UTR prediction [95]. We then checked for overlaps of de novo assembled transcripts and Iso-Seq reads with IPO323 gene models [96] using bedtools (version v2.27.1) [97]. Overlapping transcript sequences were further filtered to remove transcripts overlapping more than one gene and transcripts smaller than the predicted gene model. After filtering, the start and end position of the longest predicted transcript was considered as the 5′UTR start and 3′UTR end of each gene.

### Functional annotation of coding sequences

We retrieved existing gene annotation data for the reference strain IPO323 [96]. Genes encoding putative effectors were predicted using EffectorP v 2.0 [50]. Secondary metabolite gene clusters were predicted using antiSMASH 4.0 [51]. Genes upregulated during host infection were selected according to previous host infection transcriptome analyses [31]. Genes encoding cell wall-degrading enzymes were annotated based on matches with the Carbohydrate Active Enzymes database (CAZy) [98].

### Mapping cis-eQTL using permutations

We used QTLtools (version 1.1) [99] for transcriptomic data filtering and the mapping of eQTL. Reads mapped to gene models [96] were counted with the QTLtools –quan mode. Only reads with a minimum Phred mapping quality > 10 were kept for further analyses. Normalization of the counts was done using the –rpkm option implemented in the QTLtools –quan mode. Gene expression coefficients of variation (CV) were calculated as the standard deviation (SD) normalized by the gene expression mean value (CV = SD/mean). To determine the optimal configuration for eQTL discovery, we performed eQTL mapping with 1000 permutations and 5kb *cis* windows filtering for genes with RPKM = 0. Principal components (PCs) explaining variance at the genotype and expression level were calculated using the –PCA mode in QTLtools. To determine the number of PCs for population structure correction and technical variance in the dataset, independent eQTL analyses were performed in the *cis* –permutation mode with 1000 permutations and 5kb *cis* windows with differing numbers of PCs. The permutation *p-*values are false discovery rate (FDR) corrected to identify the top eQTL significant at a 5% FDR level. To map *cis*-eQTLs with an independent effect on gene expression, we used the QTLtools –*cis* conditional option and reported 5% FDR corrected eQTLs in association with the top variant reported from –*cis* permutation analyses. For this, we have chosen a *cis* window of 10kb equidistant from the TSS.

### Mapping trans-eQTL using permutations

To map trans-eQTLs, we used the –trans mode in QTLtools (version 1.1). We ran the full pass mode to identify the top candidates of trans-eQTLs. The full permutation scheme in the QTLtools trans analysis permutes all expression phenotypes and genetic variants excluding the window defined for *cis* variants. We ran the nominal pass with the threshold of 1e − 5 excluding variants in the window of 200 kb equidistant from the start codon of each focal gene. Multiple testing correction was performed using a full permutation scheme with 100 permutations. We have retained trans-eQTLs if the association was significant at a 5% FDR in at least 80% of the permutations. The approximation pass selected 1000 expression phenotypes randomly, permutated expression phenotypes, and tested for associations against all variants as a less stringent threshold to retain *trans-*eQTL.

### Colocalizing mapped eQTLs with GWAS-associated variants

To assess colocalization of eQTLs with GWAS signals, we used the RTC mode in QTLtools (version 1.1) [99]. We chose variants associated with phenotypes from a GWAS study based on a mapping population established from the same wheat field [100]. We analyzed GWAS for fungicide resistance, virulence, and reproduction of the pathogen for colocalization with mapped *cis*-eQTLs. We retained colocalized variants if the RTC score was > 0.9 and the linkage disequilibrium (*r*^2^) between the variants > 0.5.

### Supplementary Information

**Additional file 1: Figure S1. **Minor allele frequency distribution of SNPs and indels in the mapping population. **Figure S2.** Proportion of variance explained by the principal components based on SNP polymorphism. **Figure S3. **Left: The number of genes with eQTL reported for differing numbers of genotype (GPC) and gene expression (PH) principal components.Right: Number of genes with an eQTL mapped as a function of the required minimum percent of isolates showing gene expression. **Figure S4. **Optimization of cis window size around TSS for eQTL mapping. Left: backward nominal p-value distribution of eQTLs mapped spanning a 25kb window centered on the TSS. Right: number of cis-eQTLs reported with a window size of 25kb centered on the TSS. **Figure S5. **Number of cis-eQTLs with decreasing effect on expression from rank 1 to rank 5. **Figure S6. **SnpEeff annotation of cis-eQTLs and their effect size. There was no indel-eQTL reported as synonymous variant. **Figure S7. **Number of significant SNPs associated with different phenotypic traits in the mapping population.

Additional file 2**: Table S1. **Chromosomal location and statistics of all *cis*-eQTLs mapped in the mapping population. **Table S2. **Chromosomal location and statistics of *trans*-eQTLs identified by approximate pass. **Table S3**. Chromosomal location and statistics of *trans*-eQTLs identified by full pass. **Table S4.** Summary of virulence and fungicide sensitivity-associated genes mapped with trans-eQTLs association.

## Data Availability

All sequence data is deposited on the NCBI Short Read Archive under the BioProject accession number PRJNA650267 (https://www.ncbi.nlm.nih.gov/bioproject/PRJNA650267) [101].
