# Supplementary material for: Genome-wide expression QTL mapping reveals the highly dynamic regulatory landscape of a major wheat pathogen
Source: BMC Biol. 2023 Nov 20;21:263. doi: 10.1186/s12915-023-01763-3 (PMC10658818; doi:10.1186/s12915-023-01763-3)

Supplementary Figure S1

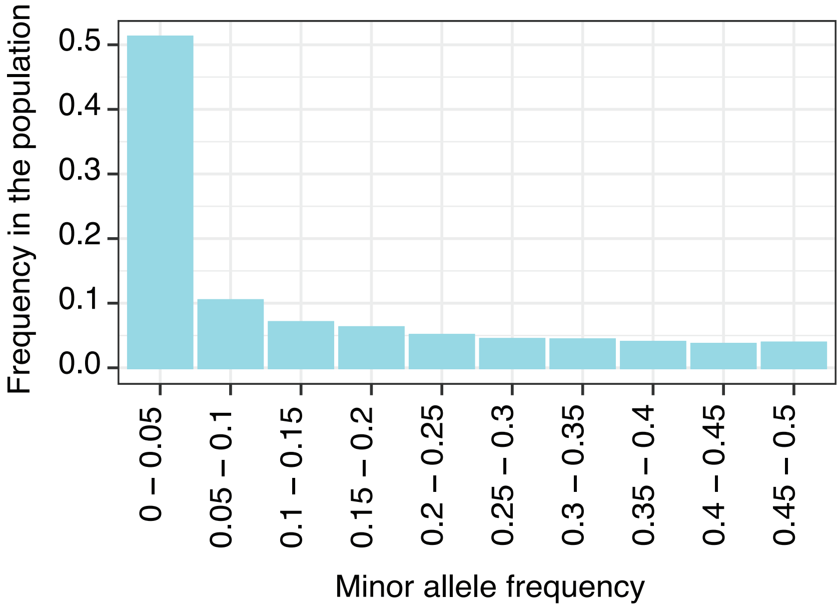

Supplementary Figure S2

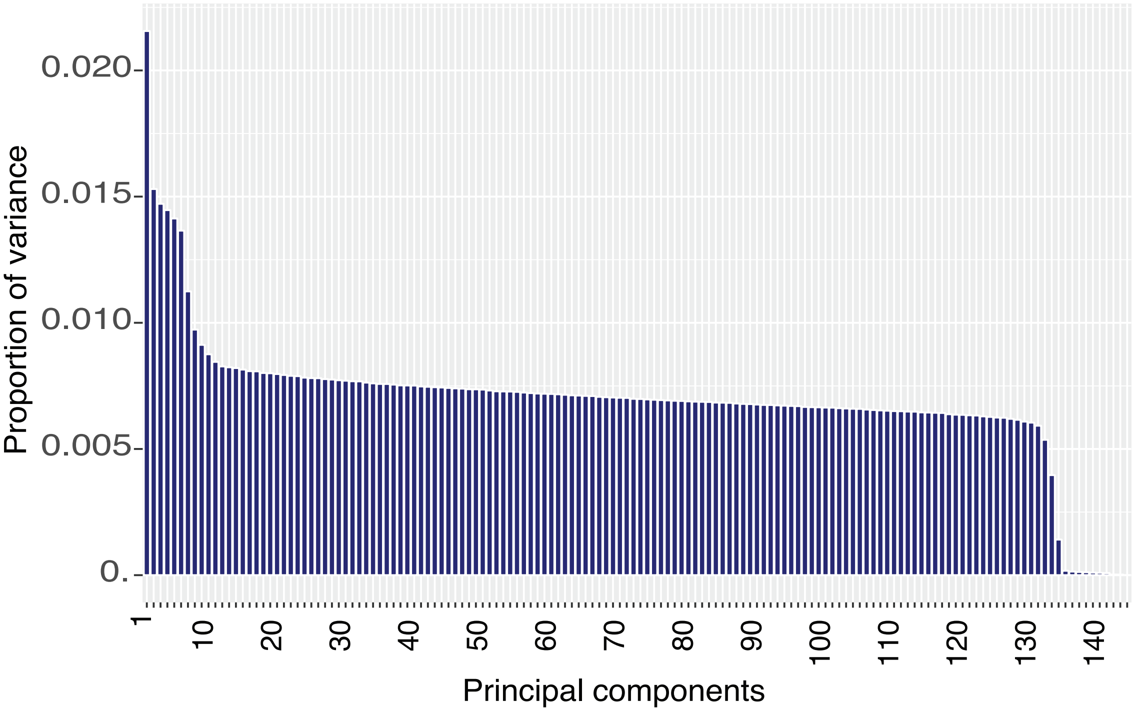

Supplementary Figure S3

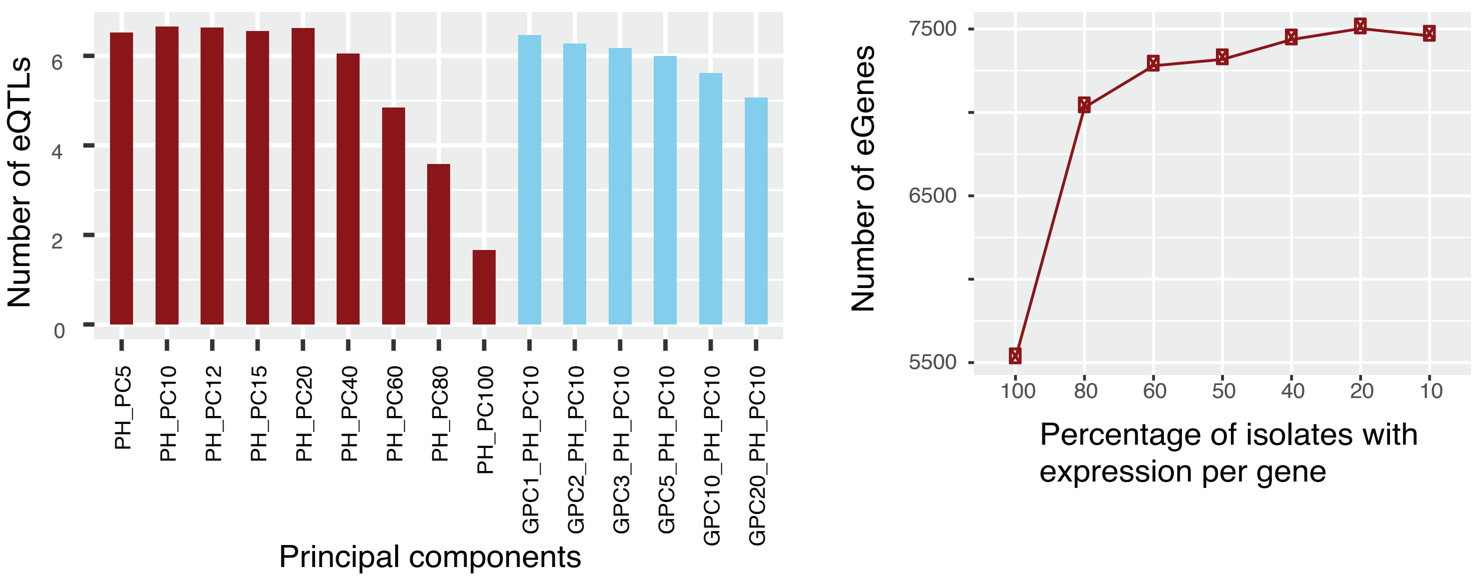

Supplementary Figure S4

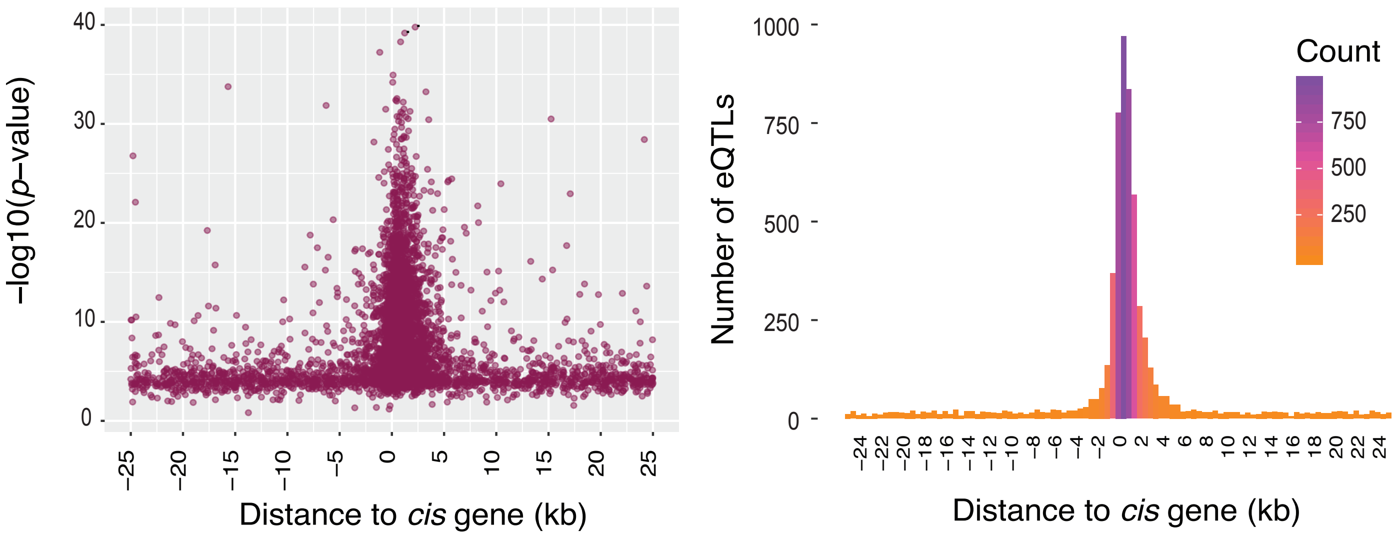

Supplementary Figure S5

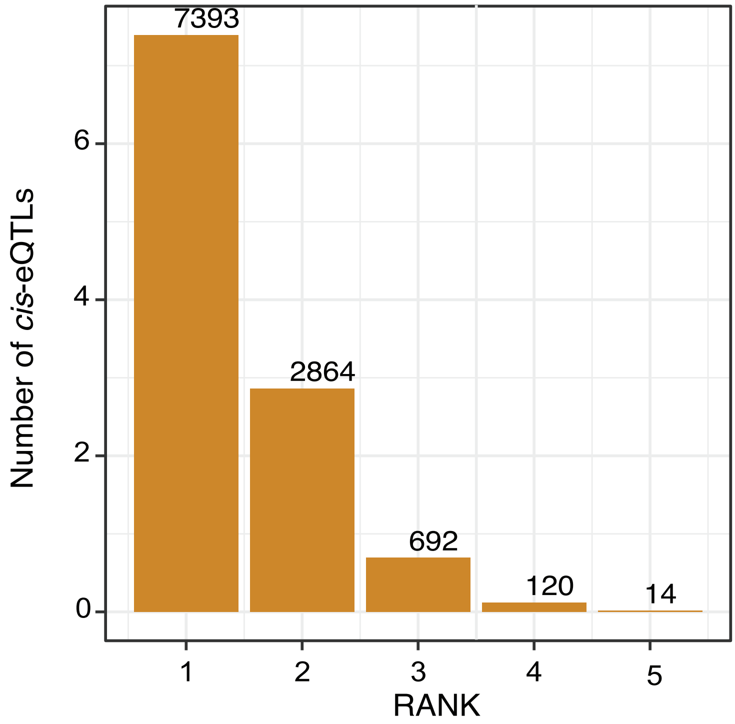

Supplementary Figure S6

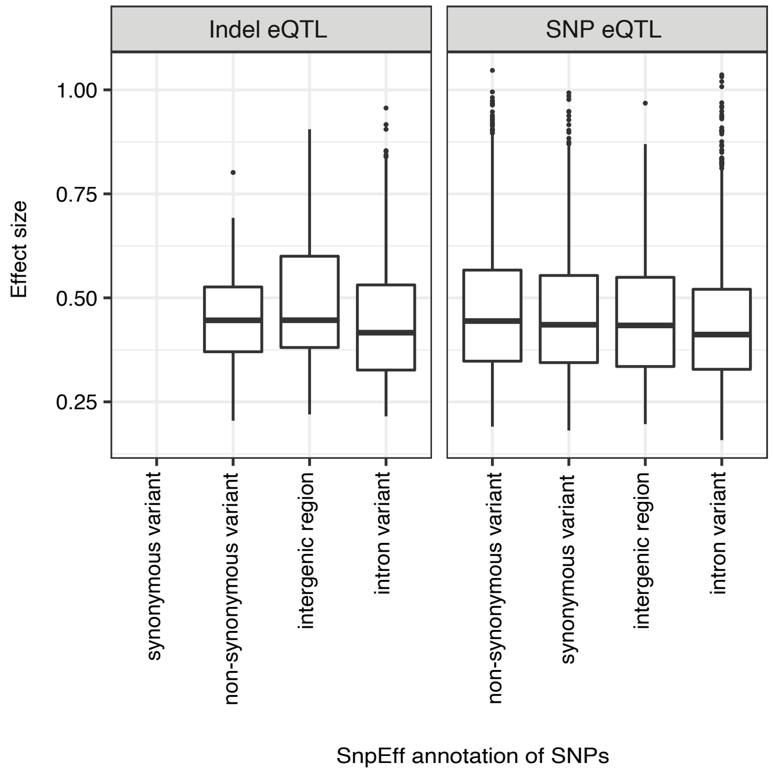

Supplementary Figure S7

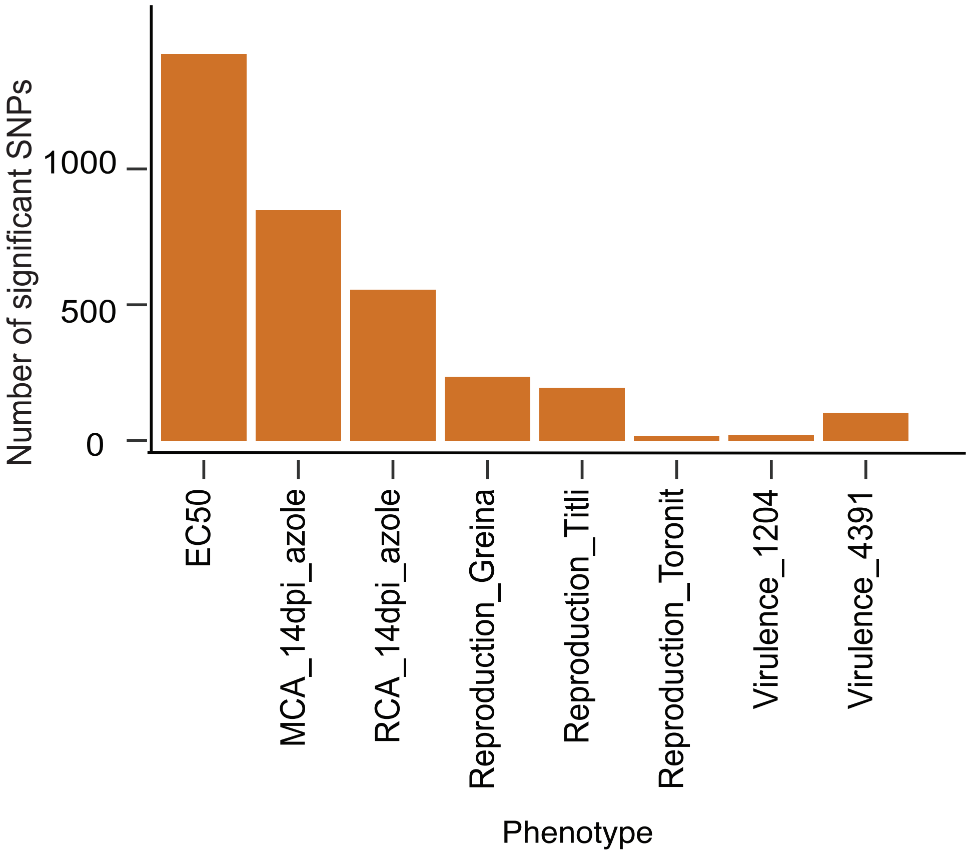

Supplement: Supplementary file 1 — Additional file 1: Figure S1. Minor allele frequency distribution of SNPs and indels in the mapping population. Figure S2. Proportion of variance explained by the principal components based on SNP polymorphism. Figure S3. Left: The number of genes with eQTL reported for differing numbers of genotype (GPC) and gene expression (PH) principal components.Right: Number of genes with an eQTL mapped as a function of the required minimum percent of isolates showing gene expression. Figure S4. Optimization of cis window size around TSS for eQTL mapping. Left: backward nominal p-value distribution of eQTLs mapped spanning a 25kb window centered on the TSS. Right: number of cis-eQTLs reported with a window size of 25kb centered on the TSS. Figure S5. Number of cis-eQTLs with decreasing effect on expression from rank 1 to rank 5. Figure S6. SnpEeff annotation of cis-eQTLs and their effect size. There was no indel-eQTL reported as synonymous variant. Figure S7. Number of significant SNPs associated with different phenotypic traits in the mapping population. [file 12915_2023_1763_MOESM1_ESM.pdf]
